# A Systematic Review of Intravenous β-Hydroxybutyrate Use in Humans – A Promising Future Therapy?

**DOI:** 10.3389/fmed.2021.740374

**Published:** 2021-09-21

**Authors:** Hayden White, Aaron J. Heffernan, Simon Worrall, Alexander Grunsfeld, Matt Thomas

**Affiliations:** ^1^Department of Intensive Care Medicine, Logan Hospital, Meadowbrook, QLD, Australia; ^2^School of Medicine, Griffith University, Southport, QLD, Australia; ^3^Department of Biochemistry and Molecular Biology, Faculty of Science, School of Chemistry and Molecular Biosciences, The University of Queensland, Brisbane, QLD, Australia; ^4^Department of Neurosciences, Eastern Virginia Medical School, Sentara Healthcare, Norfolk, VA, United States; ^5^Department of Intensive Care Medicine, North Bristol NHS Trust, School of Physiology, Pharmacology and Neuroscience, University of Bristol, Bristol, United Kingdom

**Keywords:** ketones, acetoacetate, beta-hydroxybutyrate (BHB), ketosis, intravenous

## Abstract

Therapeutic ketosis is traditionally induced with dietary modification. However, owing to the time delay involved, this is not a practical approach for treatment of acute conditions such as traumatic brain injury. Intravenous administration of ketones would obviate this problem by rapidly inducing ketosis. This has been confirmed in a number of small animal and human studies. Currently no such commercially available product exists. The aim of this systematic review is to review the safety and efficacy of intravenous beta-hydroxybutyrate. The Web of Science, PubMed and EMBASE databases were searched, and a systematic review undertaken. Thirty-five studies were included. The total beta-hydroxybutyrate dose ranged from 30 to 101 g administered over multiple doses as a short infusion, with most studies using the racemic form. Such dosing achieves a beta-hydroxybutyrate concentration >1 mmol/L within 15 min. Infusions were well tolerated with few adverse events. Blood glucose concentrations occasionally were reduced but remained within the normal reference range for all study participants. Few studies have examined the effect of intravenous beta-hydroxybutyrate in disease states. In patients with heart failure, intravenous beta-hydroxybutyrate increased cardiac output by up to 40%. No studies were conducted in patients with neurological disease. Intravenous beta-hydroxybutyrate has been shown to increase cerebral blood flow and reduce cerebral glucose oxidation. Moreover, beta-hydroxybutyrate reduces protein catabolism and attenuates the production of counter-regulatory hormones during induced hypoglycemia. An intravenous beta-hydroxybutyrate formulation is well tolerated and may provide an alternative treatment option worthy of further research in disease states.

## Introduction

3-beta-Hydroxybutyric acid (beta-hydroxybutyrate; BHB), acetoacetic acid (acetoacetate; AcAc), and acetone (Supplementary Figure 1) are collectively referred to as “ketone bodies.” In humans, AcAc and BHB accumulate when carbohydrate availability is low, due to the oxidation of fatty acids released from stores in adipose tissue to serve as an alternative energy source for cellular respiration (Supplementary Figure 2). The predominant ketone bodies, AcAc, and BHB are energy-rich and can be used to export usable energy to extra-hepatic tissues. This means that when blood glucose concentrations are low, the use of ketone bodies spares glucose (1, 2) for use by tissues such as erythrocytes and the brain that are obligate glucose users. Under these conditions the large amounts of fatty acids being released from the adipose stores cannot, unlike most other tissues, be directly used by the brain to generate energy. However, the ketone bodies generated from the excessive production of acetyl-CoA in the liver, can be used as an alternative source of energy, and can provide about 60–70% of the brain's energy requirements during extended starvation.

With this in mind, ketosis is usually associated with a pathological state, such as diabetic ketoacidosis, starvation or alcohol intoxication. Moreover, the ratio of acetoacetate to BHB (arterial ketone body ratio) reflects the efficiency of cellular metabolism (mitochondrial redox state) (3–5), and therefore can be viewed as a concerning marker of cellular hypoxia. Despite this, the intentional induction of ketosis with prolonged dietary modifications, such as ketogenic diets, may have a therapeutic role. For example, the use of such diets in two recent randomized-controlled trials showed improved seizure control in epileptic children. Ketogenic diets have also been examined in other pathological conditions including heart disease, neurological disorders such as Alzheimer disease, Parkinson disease, motor neuron disease, stroke, traumatic brain injury and migraine (6–11).

Importantly, the role of therapeutic ketosis in acute pathological states such as traumatic brain injury, stroke, heart failure and other diseases has been less extensively investigated due to the lack of a commercially available supply of intravenous BHB. Rapid induction of ketosis with intravenous BHB administration may have a therapeutic role in optimizing cellular respiration in disease states. The purpose of this systematic review is to examine the potential safety, pharmacokinetics and physiological outcomes of patients receiving intravenous BHB formulations. Whereas several reviews on ketone bodies and their utility as therapeutic agents have been published the authors believe this to be the first systematic review to focus solely on the use of BHB as an intravenous therapeutic agent.

## Methods

### Search Strategy

Studies were retrieved from the PubMed, Web of Science, and EMBASE databases. The following search phrase was used: [(keton^*^) OR (hydroxybutyrate^*^) OR (beta-hydroxybutyr^*^) OR (3-hydroxybutyr^*^) OR (sodium-hydroxybutyr^*^)] AND [(intraven^*^) OR (infusi^*^) OR (intra-veno^*^)] and limited using the relevant study filter to include only human participants where possible. Published studies from 1946 until the 27th of October 2020 were included. A bibliography search of included articles identified for full text review was also performed. Other articles were identified from a search of the author’s extensive records.

### Inclusion and Exclusion Criteria

Published peer-reviewed manuscripts were included if they included human subjects who received any intravenous formulation of BHB. Articles including animals, subjects receiving other ketone formulations, or administration of BHB by a route other than intravenous were excluded. Studies with insufficient description of BHB administration were also excluded. Articles written in a language other than English were excluded. Articles for full reference review and data extraction were selected based on the abstract of articles identified in the search. A summary of the review is presented in the Preferred Reporting Items for Systematic Reviews and Meta-Analyses (PRISMA) flow chart (Supplementary Figure 3).

### Data Extraction

The following study details were extracted, where available, from the included studies: study participant demographic details (population type, number of participants, weight and body mass index [BMI]), BHB formulation, fasting status, use of insulin, and total dose of BHB. Outcome variables extracted included BHB concentration, any relevant study outcome variable, and any adverse events experienced during the study.

Article identification, evaluation, and data extraction were performed by two independent reviewers (AH and HW). When disagreements occurred, resolution was mediated by consultation with other authors.

## Results

### BHB in Cardiac Disease

Animal studies associating ketone administration with improved cardiac performance has stimulated interested in human studies. Results for studies involving cardiovascular endpoints are summarized in Table 1. Two studies were identified. Gormsen et al., demonstrated a 75% increased myocardial blood flow and decreased glucose uptake in healthy participants following intravenous ketone administration (13). More recently, Nielsen et al. investigated the effects of BHB infusions on patients with chronic heart failure (New York Heart Association Classification II–III) with reduced ejection fraction (14). Cardiac output increased by 40% (2.0 ± 0.3 L/min) (*p* < 0.001), left ventricular ejection fraction 8%, stroke volume, heart rate and mixed venous oxygen saturation all demonstrated significant increases while systemic venous resistance decreased by 30%. Mean arterial pressure remained stable throughout. Moreover, similar to the Gormsen et al. study, myocardial blood flow also increased in this patient population. A follow up study where BHB concentrations were slowly increased to ~50% of the previous study also confirmed an increase in cardiac output (1.2 ± 0.1 L/min; *P* < 0.001) although less than the initial result. They concluded that BHB administration produces a dose-dependent increase in cardiac output (which are unlikely to be related to myocardial free fatty acid uptake) and that cardiac effects can be seen at physiological concentrations. BHB appeared safe with only two episodes of nausea and one of asymptomatic ventricular tachycardia (known condition) (12).

**Table 1:** Ketone studies investigating cardiac outcomes.

**Year**	**Author**	**Study**	**Population**	**No. participants**	**Formulation**	**Fasting**	**Intervention**	**Total average BHB dose**	**Insulin used**	**Findings overview**	**Concentration BHB**	**ADR**
1993	Vanoverschelde et al. (12)	Cross-over	Healthy	6	Dextro-3-OHB 576 mM.	Fasting	40 μmol/kg/min for 20 min followed by 17 μmol/kg/min		No	Decreased ^11^C-Palmitate myocardial uptake indicating reduced fatty acid oxidation.	0.06 ± 0.05 to 1.15 ± 0.30 mM	Nil noted
2017	Gormsen et al. (13)	Randomized placebo-controlled single blinded crossover	Healthy	8	Racaemic Na-3-OHB 7.5%	Fasting	0.18 g/kg/h for 6.5 h		Yes - EG	Increased myocardial blood flow in all coronary artery vascular territories (~75%) with an increased HR potentially associated with alkalosis and reduced potassium. Myocardial glucose uptake halved.	<50 microM to 3.78 ± 0.47 mM post-infusion	Nil noted
2019	Nielsen et al. (14)	Randomized placebo-controlled single blinded crossover	HFrEF NYHA Class II-III + Healthy	Study 1: 24/10 HFrEF/controls; Study 2: 8 HFrEF	Racaemic Na-3-OHB 7.5% + KCl 60 mM	Fasting	Study 1: 0.18 g/kg/h for 3h; Study 2: 0.045 g/kg/h for 2 h then separately 0.09 g /kg/h for 2h; Study 3: 0.18 g/kg/h for 3h		Yes - EG	Dose dependent increase in CO in patients with HF, potentially related to decreased SVR.	Study 1: 0.4 ± 0.3 mM baseline to 3.3 ± 0.4 mM after 3 h infusion. Study 2: <0.1 mM to 0.7 ± 0.1 mM following 0.045 g/kg/h; 1.6 ± 0.3 mM following 0.09 g/kg/h. Study 3: 0.4 ± 0.3 mM to 3.4 ± 0.6 mM.	Asymptomatic VT in one patient with a history of VT. Two episodes of nausea (one during placebo, other following infusion of 3-OHB).

*ADR, adverse drug reaction; BHB, beta-hydroxybutyrate; CI, continuous infusion; CO, cardiac output; EG, euglycaemic clamp; FFA, free fatty acids; NEFA, non-esterified fatty acids; GFR, glomerular filtration rate; GH, growth hormone; HFrEF, heart failure with reduced ejection fraction; HR, heart rate; NYHA New York Heart Association; SVR, systemic vascular resistance; T1DM, type 1 or insulin-dependent Diabetes mellitus; T2DM, type 2 or non-insulin-dependent Diabetes mellitus; VT, ventricular tachycardia*.

*Data presented as mean ± standard error*.

### Ketones and the Brain

All reviewed studies examined the impact of intravenous BHB on a variety of clinical and physiological endpoints (Table 2). No publications could be identified examining intravenous BHB in individuals with neuropathology.

**Table 2:** Ketone studies investigating central nervous system outcomes.

**Year**	**Author (Ref)**	**Study**	**Population**	**No. participants**	**Formulation**	**Fasting**	**Intervention**	**Total average BHB dose**	**Insulin used**	**Findings overview**	**Concentration BHB**	**ADR**
1996	Hasselbalch et al. (15)	Randomized crossover	Healthy	8	Racaemic BHB 55 mg/mL; pH 7.1	4-5 h post morning meal	4-5 mg/kg/min for 3-3.5 h		No	Global increase in cerebral blood flow by 39%. Cerebral glucose metabolism reduced by 33% with a corresponding reduction in oxygen use for glucose metabolism from 97% to 74% during hyperketonaemia.	0.31 ± 0.17 mM to 2.16 ± 0.42 mM post-infusion	Nil noted
2001	Pan et al. (16)	Case-series	Healthy	6	Dextro-BHB 200 mM; pH 7.1	Fasting	80 μmol/kg/min followed by 20 μmol/kg/min for 75 min		No	Cerebral ketone uptake may be increased during a fasting state.	0.20 ± 0.10 mM to 2.12 ± 0.30 mM post-infusion	Nil noted
2002	Pan et al. (17)	Case-series	Healthy	4	Dextro-BHB 200 mM; pH 7.1	Fasting	16.7 mL/min for 20 min followed by 22 μmol/kg/min for 120 min		No	Rapid entry of BHB into the brain with a distribution similar to glucose.	Plasma post infusion: 2.25 ± 0.24 mM. Brain post infusion: 0.18 ± 0.06 mM.	Nil noted
2002	Blomqvist et al. (18)	Placebo-controlled single-blinded	T1DM + Healthy controls	6 T1DM/6 Controls	Racaemic BHB	Fasting	6 mg/kg/min over 20 min followed by 3 mg/kg/min continuous infusion for up to 70 min		For T1DM patients only: maintain BGL between 6 and 12 mmol/L.	No difference in uptake rate was observed between T1DM and Healthy controls. Rate-limiting step for cerebral ketone metabolism is transfer from the blood to brain.	Post-infusion: T1DM 1.28 ± 0.31 μmol/mL; Healthy 0.98 ± 0.33 μmol/mL	Nil noted
2018	Svart et al. (19)	Randomized placebo-controlled crossover	Healthy	9	Racaemic Na-3-OHB 75 g/L	Fasting	0.22 g/kg/h 3-BHB for up to 4 h		No	Cerebral blood flow increased by 30% with a 14% reduction in cerebral glucose utilization. Oxygen consumption remained unchanged.	0.2 ± 0.02 mM to 5.5 ± 0.4 mM post-infusion	Nil noted
2020	Jensen et al. (20)	Randomized placebo-controlled double blinded crossover	T2DM	18	Racaemic Na-3-OHB 7.5%	Fasting	0.22 g/kg/h for up to 3 h		0.2 units/m^2^/min bolus over 3 min followed by 0.075 units/m^2^/min continuous infusion	Improved working memory performance in T2DM patients. No difference in global cognitive composite outcome was observed.	0.1 ± 0.0 mM to 2.4 ± 0.6 mM post-infusion	Mild headache and light-headedness.

*ADR, adverse drug reaction; BHB, beta-hydroxybutyrate; CI, continuous infusion; CO, cardiac output; EG, euglycaemic clamp; FFA, free fatty acids; NEFA, non-esterified fatty acids; GFR, glomerular filtration rate; GH, growth hormone; HFrEF, heart failure with reduced ejection fraction; HR, heart rate; NYHA New York Heart Association; SVR, systemic vascular resistance; T1DM, type 1 or insulin-dependent Diabetes mellitus; T2DM, type 2 or non-insulin-dependent Diabetes mellitus; VT, ventricular tachycardia*.

*Data presented as mean ± standard error*.

Blomqvist et al. examined the effect of acute hyperketonemia on the cerebral uptake of ketone bodies in nondiabetic subjects and insulin dependent diabetes mellitus patients (18). For both groups, cerebral utilization rates increased proportionately with plasma concentration of ketone bodies suggesting that the transfer from blood to brain was a rate limiting step in ketone body utilization. In contrast, Pan et al. published two studies using magnetic resonance imaging spectroscopy in healthy participants and demonstrated diffuse central nervous system (CNS) ketone uptake with a similar distribution to glucose within 15 min of administration, achieving peak mean CNS concentrations of ~0.18 to 0.24 mmol/L (16, 17). This rapid uptake may, in part, be related to increased cerebral blood flow given that cerebral uptake was linearly related to arterial BHB concentration (Mikkelsen et al.) indicating a first-order process such as passive diffusion (21). Hasselbalch et al. showed increased global cerebral blood flow by 39% (*p* < 0.02) (15). Moreover, in the absence of exogenous BHB, 97% of oxygen metabolism was used for oxidation of glucose, whereas during acute hyperketonemia, 74% of oxygen metabolism was due to glucose and 26% from ketone body oxidation. The overall rate of oxygen metabolism remained constant. Similar findings were identified by Svart et al. with an increased cerebral blood flow of 30% and a reduction in glucose oxidation by ~14% (19). The improvements in cerebral blood flow and altered oxygen metabolism may improve working memory in patients with Type 2 Diabetes mellitus. Jensen et al. administered a racemic formulation of BHB that was associated with a 17% improvement in working memory (although there was no change in global cognitive function) (20).

### Renal Effects

The impact of BHB on renal function has also been examined. Fioretto et al. compared the impact of BHB infusions on the glomerular filtration rate in 11 insulin dependent diabetes mellitus (IDDM) patients and 11 controls (22). Glomerular filtration increased significantly in the IDDM group only, without a change in pH or bicarbonate in any group. Conversely, there was no change in glomerular filtration following BHB administration in healthy males despite similar peak BHB concentrations (23). Wildenhoff investigated renal handling of ketones in IDDM and normal volunteers by administering BHB (1 mol/L) at varying rates and measuring renal clearances (24). They noted that at low filtration rates, BHB was completely reabsorbed, that this increased linearly with increasing filtration rate and that a maximal tubular reabsorption rate could not be demonstrated.

### Metabolic Effects

The majority of studies examining intravenous infusions of BHB were conducted to determine the metabolic effects of ketone infusions in normal and diabetic individuals (Table 3). These studies span a number of decades and vary significantly in both quality and study design.

**Table 3:** Ketone studies with metabolic outcomes.

**Year**	**Author (Ref)**	**Study**	**Population**	**No. participants**	**Formulation**	**Fasting**	**Intervention**	**Total average BHB dose**	**Insulin used**	**Findings overview**	**Concentration BHB**	**ADR**
1968	Balasse and Ooms (25)	Placebo-controlled	Healthy	8 BHB / 5 Controls	Racaemic Na-3-OHB	Unclear	5 mmol/kg/h for 1.5 h		No	Ketonaemia up to 47.6 mg/100 mL resulted in a mild alkalosis (pH 7.39 to 7.48) with a 50% reduction in non-esterified fatty acids and a reduced blood glucose concentration from 84 mg/100 mL to 69 mg/100 mL, without a change in insulin concentration.		Mild headache, thirst
1975	Sherwin et al. (26)	Case-series	Obese + Healthy	9 Healthy / 10 Obese	Racaemic Na-OHB 40%	12 h, 3 day and 3–5 week fast	Loading dose twice continuous infusion dose over 20 min. Non-obese controls 3 mg/kg/min for 3 h, Obese dose 110 mg/m^2^/min for 6 h.			Healthy group: No change in insulin, pyruvate, lactate and glucagon. All patients exhibited a slight reduction in both glucose and alanine.	BHB 0.84 ± 0.09 mM after infusion. Basal obese BHB 0.12 ± 0.03 mM increasing to 0.94 ± 0.10 mM post-6 h infusion. Obese after 3-days starvation: baseline BHB 1.39 ± 0.22 mM doubled post infusion. Obese after 3-5 week fast baseline 5.72 ± 0.64 mM to peak 10.39 ± 0.91 mM.	Nil noted
1976	Wildenhoff (24)	Crossover	T1D + Healthy controls	26 T1DM / 9 Controls	Racaemic Na-3-OHB 1 M	Fasting	50 mmol over 4 min		Administered to T1DM, no doses specified.	Increased urinary excretion T1DM, normalized with insulin administration. Lower ketone elimination non-obese T2DM patients but not obese T2DM patients when compared with healthy controls. Treatment with glibenclamide or phenformin had a decreased the ketone elimination in the obese, but not non-obese T2DM patients compared with healthy controls. Decreased tissue ketone uptake in non-obese T2DM patients may mediate lower elimination.		Nil noted
1976	Sherwin et al. (27)	Crossover	T1DM + Healthy	7 T1DM / 12 Healthy	Racaemic Na-BHB	Fasting	6 mg/kg/min bolus over 20 min followed by 3 mg/kg/min over 3–4.5 h.		1 participant 1mU/kg/min	Ketone clearance decreased by 42% in T1DM. Blood glucose concentration reduced by 25% in T1DM and marginally reduced in healthy controls. Alanine concentration reduced.	T1DM BHB 0.40 ± 0.08 mM to 1.67 ± 0.11 mM. T1DM AcAc 0.11 ± 0.02 mM to 0.41 ± 0.06 mM. Healthy BHB 0.12 ± 0.02 0.82 ± 0.06 mM. Healthy AcAc 0.04 ± 0.01 to 0.24 ± 0.01 mM.	Nil noted
1980	Frølund et al. (28)	Crossover	Healthy	6	Racaemic Na-3-OHB	Fasting	8 mg/kg loading dose followed by infusion 4 mg/kg/min for 90 min. 3 subjects also had 26 mg/kg loading dose followed by 13 mg/kg/min CI	Loading dose 24.20 g and maintenance dose 78.94 g	Insulin 0.15 units/kg to induce hypoglycaemia.	Ketone infusion did not change hypoglycaemic symptoms. Ketone administration did not change catecholamine or cortisol response to hypoglycaemia.	0.1 mM to 0.45 mM at a 4 mg/kg/min BHB dose and 4.0 mM following a 13 mg/kg/min BHB dose.	Shivering and nausea in all patients receiving 13 mg/kg/min (high dose) Na-3-OHB following insulin injection.
1983	Miles et al. (29)	Crossover	Healthy	6	Racaemic Na-OHB 3 M, pH 7	Fasting	30 μmol/kg/min for 20 min followed by μmol/kg/min for 3 h. Doses based on D-enantiomer.		No	No decrease in proteolysis with reduced alanine and free fatty acids.	Total ketone body baseline 0.21 ± 0.04 mM to 2.33 ± 0.48 mM post infusion	Nil noted
1983	Quabbe et al. (30)	Crossover	Healthy	10	Racaemic Na-3-OHB 7.5%	Fasting	15 g/h for 3 h		Yes - induce hypoglycaemia	FFA suppressed during BHB infusion. Growth hormone increased. Glucagon increased by 10–20 pg/mL. Delayed rebound of FFA and GH following insulin-induced hypoglycaemia.		Nil noted
1983	Woods et al. (31)	Placebo controlled	Post-operative cholecystectomy	17 BHB / 15 Controls	Racaemic Na-3-OHB	Fasting	0.75 g/h for 3 days	54 g	No	No difference post-operative mean daily urinary nitrogen losses.		Nil noted
1986	Bratusch-Marrain et al. (32)	Crossover	Healthy	9	Racaemic-Na-OHB 4 M; pH 7.24	Fasting	30 μmol/kg/min for 20 min followed by 15 μmol/kg/min for 4 h		Yes - EG	Glucose appearance rate and metabolism were unchanged during and after BHB infusion.	109 ± 31 μM to 496 ± 81 μM post-infusion	Nil noted
1986	Christensen et al. (23)	Case-series	Healthy	7	Racaemic Na-BHB 50 g/L	Fasting	0.5 g/kg over 1 h	35.5 g	No	No change in urinary albumin excretion, GFR or blood pressure. Small increase beta-2-microglobuin excretion.	0.05 ± 0.05 mM baseline to 1.96 ± 0.53 mM	Nil noted
1986	Desir et al. (33)	Crossover	Healthy	7	Racaemic-Na-BHB 40%	Fasting	1 mmol/kg over 20 min followed by 0.01 mmolkg/min for 160 min.		No	Increased serum and urine pH, reduced potassium (4.18 ± 0.09 to 3.58 ± 0.06 mM), 35% increased urinary ammonia excretion. GFR unchanged.	53 ± 9 μmol/L to ~200 μmol/L post-infusion	Nil noted
1987	Fioretto et al. (22)	Crossover	T1DM + Healthy	11 T1DM / 11 Controls	Racaemic 3-hydroxybutyric acid/Racaemic Na-3-OHB	Fasting	40/30 μmol/kg/min for 2 h	30 μmol/kg /min: 367.2 mmol (46.3 g) T1DM and 378 mmol (47.66 g) Healthy; 40 μmol/kg/ min: 489.6 mmol (61.73 g) T1DM and 504 mmol (63.55 g) Healthy	Yes - EG	GFR increased T1DM. No change pH or bicarbonate.	Following infusion 40 μmol/kg/min healthy controls total ketone body concentration of 2.719 ± 0.163 mM and 3.089 ± 0.149 mM T1DM. Following infusion of 30 μmol/kg/min, total ketone body concentration of 0.95 ± 0.17 mM and 1.49 ± 0.27 mM in T1DM.	Nil noted
1988	Nair et al. (34)	Crossover	Healthy	13	Racaemic Na-BHB; pH 6.8–7.4	Fasting	12.5 μmol/kg/h	452.63 mmol (57.07 g)	No	Decreased glucose and free fatty acids with unchanged c-peptide, glucagon, noradrenaline and adrenaline concentrations. Decreased leucine oxidation unrelated to a change in blood pH; thereby likely promoting protein synthesis.	<0.5 mM to ~2 mM post-infusion.	Nil noted
1989	Crowe et al. (35)	Case-series	Post-operative Total Hip Replacements	6	Racaemic-Na-BHB	Fasting	6 mg/kg/min over 20 mins followed by 3 mg/kg/min for 2 h		No	Leucine concentrations and rate of appearance increased following BHB infusion. Insulin concentration not affected following BHB infusion.	Pre-op 0.06 ± 0.01 to 0.69 ± 0.16 mM post-infusion. Post-op 0.04 ± 0.01 to 0.62 ± 0.12 mM post-infusion.	Nil noted
1990	Moller et al. (36)	Cross-over	T1DM	5	Racaemic-Na-OHB; pH 6.5	Fasting	1.8 mmol/kg/h for 20 min and 0.9 mmol/kg/h for 100 min		Yes - EG	NEFA decreased in both hypo- and hyperglycaemia conditions. No change in NEFA clearance.	Pre-infusion: euglycaemia 127 (30-435) μmol/L and hyperglycaemia 137 (20-350) μmol/L. Post-infusion: euglycaemia 770 (520-955) μmol/L and hyperglycaemia 665 (450-910) μmol/L.	Nil noted
1991	Amiel et al. (37)	Randomized placebo-controlled crossover	Healthy	6	Racaemic Na-OHB 6.24–7.38 g/100 mL	Fasting	6 mg/kg/min for 20 min followed by 3 mg/kg/min.	48.1 g	Yes - EG	Following hypoglycaemia, all hormone peak concentrations (noradrenaline, adrenaline, cortisol and growth hormone) were decreased in subjects receiving BHB compared with controls.	61 ± 17 μmol/L to 580 ± 69 μmol/L post-infusion.	Nil noted
1991	Hiraide et al. (38)	Placebo-controlled	Blunt trauma	11 BHB / 9 Controls	Racaemic Na-3-OHB 20%	Unclear	25 μmol/kg/min for 3 h	247.5 mmol (31.21 g)	No	Slight decrease in non-esterified fatty acids and alanine release. Mild alkalosis for both sodium lactate and BHB infusion.	Total ketone body 210.5 ± 176.0 μmol/L to 1519.2 ± 420.6 μmol/L.	Nil noted
1991	Walker et al. (39)	Randomized crossover	Healthy	7	Racaemic Na-3-OHB 3 M; pH 7.2	Fasting	15 μmol/kg/min over 4 h		Yes - EG	Slight decrease and increase in glucose and lactate respectively. BHB did not inhibit insulin stimulated glucose uptake.	Total ketone body levels from 70 ± 4 to 450 ± 30 μM post-infusion.	Nil noted
1992	Beaufrere et al. (40)	Placebo controlled	Healthy	6 BHB / 4 Controls	Dextro-BHB 0.33 g/L; pH 6.8	Fasting	540 μmol/kg/h for 5 h		No	73% reduction FFA. Unchanged cortisol, insulin and C-peptide concentrations. No significant change leucine oxidation.	Total ketone body from 180 ± 60 to 1647 ± 275 μM	Nil noted
1993	Chiolero et al. (41)	Crossover	Healthy	6	Racaemic-Na-OHB	Fasting	20 μmol/kg/min for 3 h		No	FFA decreased. Increased bicarbonate concentration. Oxygen consumption increased 5.5%. Carbohydrate oxidation inhibited 25%.	0.02 ± 0.01 mM to 0.94 ± 0.07 mM post-infusion.	Nil noted
1994	Beylot et al. (42)	Controlled	Sepsis patients in ICU + Healthy	12 Sepsis/6 Healthy	Dextro-OHB, pH 6.8	Unclear	15 μmol/kg/min over 4 h		No	No change in pH. Slight decrease in free fatty acids, glycerol and endogenous glucose production. Mild insulin increase and leucine oxidation.		Nil noted
1994	Veneman et al. (43)	Randomized, crossover	Healthy	13	Racaemic-Na-BHB	Fasting	40 μmol/kg/min for 20 min followed by 20 μmol/kg/min		Yes - EG	BHB infusion decreased counterregulatory hormone response to hypoglycaemia with 57% reduction in epinephrine and 28% reduction in cortisol. Reduced neuroglycopenic symptoms with BHB infusion.	10 ± 5 μmol/L to 1.9 mmol/L	Nil noted
1997	Wildenhoff (24)	Case-series	T1DM + Healthy	7 T1DM/8 Healthy	Racaemic-Na-BHB; 1 M	Fasting	50 mmol followed by 0 to 2.5 mmol/min		No	Linear relationship showing increased total urinary ketone body excretion and reabsorption rate with increasing urinary filtration rate in both healthy controls and T1DM patients.	0.015 to 2.91 mM post-infusion.	Nil noted
2015	Mikkelsen et al. (21)	Case-series	Healthy	6	Dextro-OHB	Fasting	4.7 μmol/kg/min for 1 h then 9.4 μmol/kg/min for 1 h then 18.8 μmol/kg/min thereafter for 50 min.	159.89 mmol (20.16 g)	No	14% reduction glucose appearance and 37% decrease lipolytic rate. Insulin and glucagon concentrations unchanged. Cerebral OHB uptake kinetics linear, whereas skeletal muscle kinetics saturable.	Peak 1.7 mM	Nil noted
2018	Thomsen et al. (44)	Double blinded placebo-controlled crossover	Healthy	10	Racaemic Na-3-OHB 7.5%	Fasting	0.18 g/kg/h 3-OHB	101.05 g	Yes - EG	Net decrease protein loss. No effect on cytokine production.	BHB basal 115 (95% CI 20-210) to 3449 (1249-5652) mM. BHB clamp 12 (95% CI 2-22) to 3280 (95% CI 1184-5375) mM.	Nil noted
2020	Lauritzen et al. (45)	Randomized placebo-controlled crossover	Healthy	9	Racaemic-Na-OHB	Fasting	0.22 g/kg/h for 4 h		No	Insulin, glucagon and Fibroblast growth factor-21 concentrations unchanged.	0.0 ± 0.0 mM to 5.5 ± 0.4 mM	Nil noted

*ADR, adverse drug reaction; BHB, beta-hydroxybutyrate; CI, continuous infusion; CO, cardiac output; EG, euglycaemic clamp; FFA, free fatty acids; NEFA, non-esterified fatty acids; GFR, glomerular filtration rate; GH, growth hormone; HFrEF, heart failure with reduced ejection fraction; HR, heart rate; NYHA New York Heart Association; SVR, systemic vascular resistance; T1DM, type 1 or insulin-dependent Diabetes mellitus; T2DM, type 2 or non-insulin-dependent Diabetes mellitus; VT, ventricular tachycardia*.

*Data presented as mean ± standard error*.

Several studies examined the impact of IV ketone infusion on protein metabolism. Reduced protein catabolism (as determined by decreased plasma alanine concentrations and reduced leucine oxidation) and increased protein synthesis are consistent findings across studies when a BHB concentration >1 mmol/L is achieved (26, 27, 29, 34). Similar findings have been shown in patients post-surgery, or involved in a trauma, which are usually associated with a negative nitrogen balance indicating muscle and protein catabolism. Crowe et al. identified an increased leucine concentration following BHB infusion in post-operative patients while Hiraide et al. noted a decreased alanine release from muscles; both situations suggesting a *nett* reduction in protein synthesis (35, 38). Conversely, Woods et al. did not identify a difference in nitrogen balance in patients following cholecystectomy (31). Prolonged fasting (weeks) in addition to intravenous BHB increased BHB concentrations from a mean of 5.72 mmol/L – 10.39 mmol/L. Despite a supraphysiological BHB concentration, all patients had a slight reduction in glucose and alanine concentrations while there was no change in insulin, pyruvate, lactate, or glucagon.

Two investigators have examined the impact of systemic inflammation and sepsis on BHB metabolism. Thomsen et al. investigated the impact of exogenous BHB and free fatty acid supplementation on protein metabolism following lipopolysaccharide infusion in 10 healthy volunteers (44). Infusions of BHB (0.18 g/kg/h) decreased protein catabolism but did not affect cytokine production following lipopolysaccharide administration. Beylot et al. investigated the effect of BHB on protein, lipolysis, and glucose production in a group of septic patients (42). Results noted a decrease in free fatty acid and glycerol concentrations with a minor decrease in glucose endogenous production but no reduction in leucine oxidation.

Amiel examined the effect of ketone infusion on counter-regulatory hormones during induced hypoglycaemia in 6 healthy volunteers (placebo cross-over) (37). The glucose concentration threshold stimulating release of counterregulatory hormones was lower when BHB was administered to achieve a concentration of ~0.5 mmol/L. All hormone responses were reduced including noradrenaline, adrenaline, cortisol and growth hormone. Similarly, Veneman et al. noted a 57% decrease in counter-regulatory hormone concentrations and autonomic symptoms during insulin induced hypoglycaemia in healthy volunteers at BHB concentrations of 2.9 mmol/L (43). Conversely, Frølund et al. in a similar study did not identify any symptom modification from BHB administration following induced hypoglycaemia (28). Moreover, there was no difference in neuroglycopaenic symptoms, adrenaline, noradrenaline and cortisol release, despite a BHB concentration of up to 4 mmol/L. The effect of BHB appears independent of insulin stimulated glucose uptake (39). Balasse et al. evaluated the role of insulin in modifications of blood glucose and non-esterified fatty acids induced by BHB (25). β-hydroxybutyrate decreased non-esterified fatty acids by 50% while the insulin concentration remained unchanged. Similarly, Lauritzen et al. investigated the effect of acute hyperketonemia on the uptake of glucose and palmitate in abdominal organs, adipose tissue, and skeletal muscle (45). β-hydroxybutyrate infusion did not affect glucose or palmitate uptake suggesting that ketone bodies are selectively used by critical organs such as heart and brain.

## Studies With Acetoacetate

Although most investigators have utilized BHB, acetoacetate has also been studied. Nosadini et al. investigated the kidney haemodynamics after ketone body and amino acid infusions in IDDM (46). Fifteen patients with IDDM and eight controls received an infusion of acetoacetate at 25 mmol/kg/min for 3h. The total ketone bodies reached 2.2 ± 1.5 mmol/l and was similar between groups. While glomerular filtration increased, albumin excretion in IDDM patients did not. Similarly, Owen et al. administered acetoacetate to assess influence of ketone bodies on insulin and free fatty acid release and to determine rates of uptake and distribution (47). Total ketone bodies reached 4.74 ± 0.39 mmol/l. Tissue utilization rates increased with BHB concentration to a peak at 4–5 mmol/l. Free fatty acid decreased, and glucose was unchanged. Frey et al. administered acetoacetate to eight normal subjects at 1.9 mmol/min x 1.73 m^2^ for 210 min and evaluated changes during exercise (48). Acetoacetate peaked at 2.54 ± 0.16 mmol/l. Free fatty acid and glucose declined as did ketones during exercise.

### BHB Dose, Pharmacokinetics and Adverse Events

Of studies examined, only eight provided average patient weight allowing for estimation of total ketone infused during study period. Total BHB administered ranged from ~30–101 g (in the study by Thomsen et al.) (44). The racemic isomer was utilized in most studies. Most authors reported on BHB and acetylacetate concentrations rather than total dose. Furthermore, plasma concentrations of BHB ranged from < 1 mmol/l to > 5 mmol/l with the highest 10.39 ± 0.91 mmol/l recorded in the study by Sherwin et al. in fasted patients (27). Many studies demonstrated a rapid increase in plasma BHB with some reaching concentrations of >1 mmol/l within 15 min. Sherwin et al. investigated the effect of diabetes mellitus on ketone body removal rates, comparing 12 healthy participants with seven IDDM volunteers (27). Baseline ketone body concentrations were higher in IDDM then controls with an approximately twofold higher BHB peak concentration in patients with IDDM. Clearance of ketones for IDDM was 42% below healthy participants. They also noted a 25% decrease in glucose and ketone disposal in IDDM.

A common concern of exogenous ketone supplementation is the risk of ketoacidosis, especially in diabetics (49, 50). There are no reports in the literature of diabetic ketoacidosis (DKA) resulting from intravenous ketone supplementation (although some trials excluded diabetic patients) which appears to improve glucose control in the short term. Furthermore, data from trials using enteral ketone supplementation to induce ketosis suggest the incidence of DKA is exceedingly low and limited to case reports. Bolla et al. reviewed the literature pertaining to ketogenic diets in diabetics, both type 1 and 2, and suggests that ketogenic diets were safe although close monitoring for DKA was necessary (51). Case reports from children with diabetes and epilepsy managed with a ketogenic diet suggest the treatment was safe provided patients are closely monitored. The few case reports of patients developing DKA secondary to ketogenic diets appears to be limited to individuals with undiagnosed type 1 IDDM who initiated the ketogenic diets and subsequently developed DKA (52–54).

Overall, it appears that the ketone infusions were well tolerated with minimal reported side effects. Common reported issues included a tendency toward alkalemia secondary to an increase in bicarbonate (although few reported pH >7.50), glucose concentrations tended to decrease although remained within the normal range, protein in the form of alanine decreased and lactate increased (but remained in the normal range). BHB appears to be readily taken up by the brain and leads to increase blood flow including cerebral, renal, and cardiac. Human studies are consistent with animal studies which demonstrated significant increase in cardiac function with BHB infusions. Overall, intravenous ketone administration appears safe and well tolerated.

## Discussion

Our review has highlighted that the intravenous administration of BHB is able to rapidly achieve supraphysiological BHB concentrations and appears safe, although the total number of study participants remains small. Most physiological effects appear to occur at systemic BHB concentrations exceeding 2 mmol/L, which is achievable after a dose of ~0.2 g/kg/h. However, no pharmacokinetic study has been performed in patients receiving intravenous BHB.

### Why Consider an Intravenous Formulation?

Until recently, generating significant ketosis in adults was difficult and required either prolonged fasting or a ketogenic diet. As the increase in plasma ketones via these techniques could take several days, neither option was useful in either the sub-acute or acute setting where a rapid increase in ketones was required (eg. Status epilepticus, acute brain injury, cardiac failure etc.). Given the encouraging evidence, both theoretical and experimental, that ketone supplementation may improve cerebral energetics post-acute brain injury, an IV formulation could likely provide a means of rapidly and predictably increasing plasma ketone concentrations. In 2005 a review paper was published examining an intravenous ketone formulation containing the sodium salt of dextro-BHB (KTX 0101) produced by a company called KetoCytonyx (55). The company claimed to have undertaken a Phase I trial where KTX 0101 was administered to 20 healthy volunteers in whom it was reportedly well tolerated with no evidence of serious side effects. The intention of KetoCytonyx was to administer their formulation to patients undergoing cardiopulmonary bypass to preserve the metabolic function of neurons during transient ischemia caused by microemboli generated during the procedure in the hope of reducing adverse neurological and psychiatric outcomes. However, the study was never published in a peer review journal and no further development of the product has taken place.

Intravenous administration should theoretically increase both plasma and cerebral ketone concentrations, with minimal adverse reactions. This was demonstrated by Neilsen et al. who were able to increase plasma BHB concentrations to above 3 mmol/l within 3h of initiating an infusion of a 7.5% Na-3-OHB solution (14). This was also demonstrated by Pan et al. who noted an increase in plasma BHB from 0.20 ± 0.10 mmol/l to 2.12 ± 0.30 mmol/l and cerebral tissue BHB from 0.16 ± 0.07 mmol/l to 0.24 ± 0.04 mmol/l following a 75 min infusion of dextro-BHB (16, 17). This has also been demonstrated in animal models where a 6h infusion of hypertonic solution containing 120 mmol/L BHB increased plasma BHB from 0.26 ± 0.05 mmol/l to 0.70 ± 0.27 mmol/l with a proportional increase in cerebral tissue BHB, which was related to the concentration of BHB in the solution.

The study by Neilsen et al. is one of the only studies examining ketone supplementation in a disease state in humans (14). Intravenous BHB was demonstrated to significantly improve the ejection fraction in patients with heart failure in a dose-dependent manner. This was the most recent study to demonstrate a clear clinical benefit for intravenous ketone administration and the first to demonstrate that modulation of circulating ketone levels may represent a novel treatment principle in patients with heart failure. Importantly, these benefits were noted with minimal side effects suggesting intravenous BHB is safe, at least in the short term. However, few studies have investigated prolonged administration of exogenous intravenous ketones.

Although ketone infusions modulate several physiological endpoints, little is known about their impact in disease states. Certainly, there is more data from enterally induced ketosis which suggests that supplementation is effective and beneficial, but ketosis secondary to intravenous administration needs further research. Also, unlike oral supplementation which has been studied over days and weeks, the longest duration reported for intravenous ketones was by Woods et al. who administered BHB for 3 days (31). Most other reports use infusions of less than 6h. This is significant as long-term infusion may lead to an increased pH. This would depend on a number of factors such as the BHB concentration in solution and total dose administered. Neilsen et al. noted an increase in plasma pH in the ketone (7.46 ± 0.03) compared with saline (7.42 ± 0.06) groups (*p* = 0.001) and here the infusion was only for 3h. Further study is necessary to examine pH changes following longer infusions.

### Why Hasn't an Intravenous BHB Formulation Been Developed?

There are several reasons why an intravenous formulation has yet to be commercially developed. First, there is a paucity of data regarding ketone pharmacokinetics and pharmacodynamics. The complexity is increased given that there are two stochiometric formulations, the dextro- and levo- isomers. Most prior studies have made their formulation utilizing a racemic mixture of BHB (containing a 50:50 mixture of dextro and levo isomers). There is some evidence that levo-BHB is not metabolized significantly into energy intermediates and is slowly excreted in the urine, therefore a pure dextro-BHB containing formulation would likely be the most effective. Moreover, the specific target concentration for optimal efficacy in disease states is unknown. Early reports by Cahill et al. based on physiological studies suggested that a plasma concentration of >4 mmol/l was required to provide adequate cerebral levels based on evidence from starvation rather than intervention studies (56). More recently this has been questioned as the correlation between plasma concentration and efficacy is poor. The majority of clinical research comes from the pediatric epilepsy population where data suggests that plasma concentration does not correlate with efficacy and often anti-convulsant effects continue long after ketogenic diet is ceased (57). Therefore, the optimal dose and formulation BHB concentration is unknown, although a potential starting concentration may be to target >2 mmol/L.

Second, the optimal formulation is unknown. Prior studies have included a large variety of ketone concentrations of different solutions. Nielsen et al. utilized a 7.5% racemic solution, but the infusions ran for a maximum of 3h and as previously noted; it is likely that this solution would be alkalinizing if infused for prolonged periods (14). A 70 kg patient would have received ~38 g of BHB over the 3h period (although authors did not report on weight). This is in contrast with the approximate 49 g received by participants in the Clarke et al. study for a patient of average weight (58). Animal studies suggest administration of <1g/kg per day is safe. Potential side effects include a dose dependent alkalosis, hypernatraemia and fluid overload.

Third, the cost of producing an intravenous ketone formulation using commercially available BHB salts is prohibitively high, particularly with the high doses of BHB that may be required (>0.2 g/kg/h). The cost is further increased if a specific enantiomer (dextro-BHB) is desired. Lastly, given the high cost of producing intravenous ketone formulations, there is a paucity of published literature in disease states that has likely deterred appropriate investment in this potential drug treatment. Less expensive production methods are possible by hydrolysis of the poly-D-BHB obtained by fermentation of *Alcaligenes eutrophus* although the regulatory hurdles would likely be restrictive. Another option would be to produce an intravenous formulation using the ketone ester. There are however concerns that this may not be effective as part of the metabolism of the ketone ester takes place in the gut. Derochers et al. demonstrated that an intravenous formulation of (R, S)-1,3-butanediol acetoacetate ester given parenterally to pigs, produced total ketone levels of 5 mmol/L without deleterious side effects (59). No studies in humans exist.

Taken together, there is a compelling argument for the development of an intravenous ketone formulation. Certainly, there is enough research to suggest that intravenous delivery of ketones is safe for short treatment durations and leads to a rapid increase in plasma ketone concentrations. Potential advantages of an intravenous formulation include the ability to administer to sedated patients and those with swallowing abnormalities, as well as dose titration to the desired plasma concentration. Currently the cost of producing an intravenous formulation is restrictive but as noted, other sources of BHB could be utilized to reduce costs of production. The production of a commercially available product could potentially lead to a rapid increase in research and potential applications of this useful energy substrate.

### Are There Enteral Alternatives?

While generally effective at inducing ketosis over time, the ketogenic diet and other traditional enteral ketogenic formulations may have limited utility in the setting of acute neurological disorders such as stroke or traumatic brain injury. Recently, the development of oral ketone esters, currently marketed as HMVN or KetoneAid, has led to the potential to increase ketones rapidly via the enteral route. Pharmacokinetic studies in healthy volunteers of ketone esters by Clarke et al. demonstrated peaks of 3.30 mmol/l at 2.5h post ingestion (58). This is comparable to the peak concentrations noted by Neilsen et al. following intravenous administration (14). Interestingly, the oral ketone ester was able to reach similar concentrations within a similar time scale but was associated with significant gastrointestinal side-effects. Although subjects were fasted for 10h prior to the study, evidence suggests that ketone ester supplementation leads to increased BHB concentrations even in the non-fasted state. The oral ketone ester supplements are registered as food, not drugs, and marketed as a performance enhancer for athletes. As such, there are no clinical trials in humans with cerebral or cardiac pathology. The only human study published to date is a case report on a patient with severe Alzheimer's Disease whose cognitive function was significantly improved by the administration of a ketone monoester, 28.7g thrice-daily for 20 months. This was well tolerated with no severe adverse reactions.

## Conclusion

Intravenously administered BHB can rapidly increase BHB concentrations to >2 mmol/L, while only minimally reducing glucose concentrations. Overall, intravenous BHB appears safe. Further research is required to determine the likely optimal dose and future clinical studies to ascertain the potential benefit of BHB in specific disease states such as neurological conditions.

## Data Availability Statement

The original contributions presented in the study are included in the article/Supplementary Material, further inquiries can be directed to the corresponding author.

## Author Contributions

HW conceptualized the review, reviewed articles for inclusion, performed the second review of data, and assisted with manuscript writing. AH designed the search strategy, reviewed articles for inclusion, performed the initial data extraction, and assisted with manuscript writing. SW, AG, and MT assistance with review design and manuscript writing. All authors contributed to the article and approved the submitted version.

## Conflict of Interest

The authors declare that the research was conducted in the absence of any commercial or financial relationships that could be construed as a potential conflict of interest.

## Publisher's Note

All claims expressed in this article are solely those of the authors and do not necessarily represent those of their affiliated organizations, or those of the publisher, the editors and the reviewers. Any product that may be evaluated in this article, or claim that may be made by its manufacturer, is not guaranteed or endorsed by the publisher.

## Abbreviations

AcAc: acetoacetate
acetyl CoA: coenzyme A
ADR: adverse drug reaction
BHB: β-hydroxybutyrate
BMI: body mass index
CI: continuous infusion
CO: cardiac output
DKA: diabetic ketoacidosis
EG: euglucemic
FFA: free fatty acids
GFR: glomerular filtration rate
CNS: central nervous system
GH: growth hormone
HFrEF: heart failure with reduced ejection fraction
HR: heart rate
NYHA: New York Heart Association
IDDM: insulin dependent diabetes mellitus
NEFA: non-esterified fatty acids
T1DM: type 1 or insulin-dependent Diabetes mellitus
T2DM: type 2 or non-insulin-dependent Diabetes mellitus
SVR: systemic vascular resistance
VT: ventricular tachycardia.
