# Supplementary material for: A Systematic Review of Intravenous β-Hydroxybutyrate Use in Humans – A Promising Future Therapy?
Source: Front Med (Lausanne). 2021 Sep 21;8:740374. doi: 10.3389/fmed.2021.740374 (PMC8490680; doi:10.3389/fmed.2021.740374)

Figure 2: The metabolism of ketone bodies**.** The production of ketone bodies is referred to as ketogenesis, and their breakdown is called ketolysis. The ultimate fate of ketone bodies is generally being used in energy production, but they are sometimes used in biosynthesis. AcAc, acetoacetic acid; BHB, b-hydroxybutyrate; CoASH, unconjugated coenzyme A; HMG-CoA, 3-hydroxymethylglutaryl-CoA.

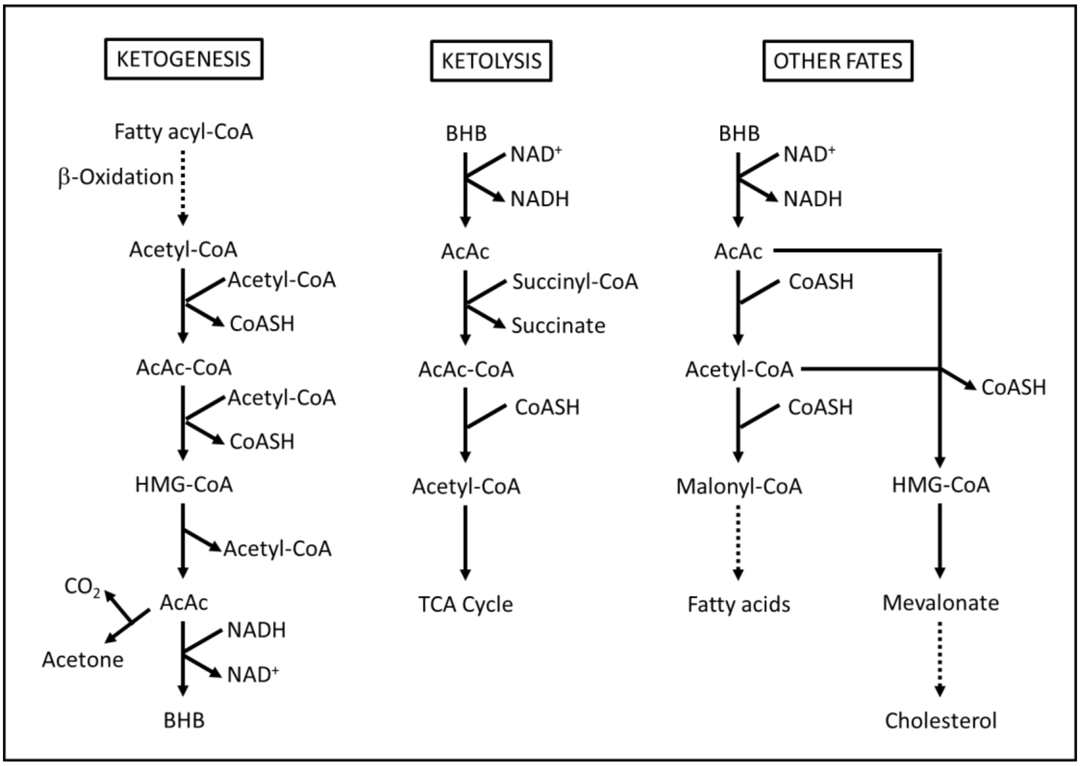

Supplement: Supplementary file 1 [file Data_Sheet_1.PDF]
