# Supplementary material for: A Systematic Review of Intravenous β-Hydroxybutyrate Use in Humans – A Promising Future Therapy?
Source: Front Med (Lausanne). 2021 Sep 21;8:740374. doi: 10.3389/fmed.2021.740374 (PMC8490680; doi:10.3389/fmed.2021.740374)

Figure 1: The chemical structures of the three compounds collectively referred to as 'ketone bodies'.

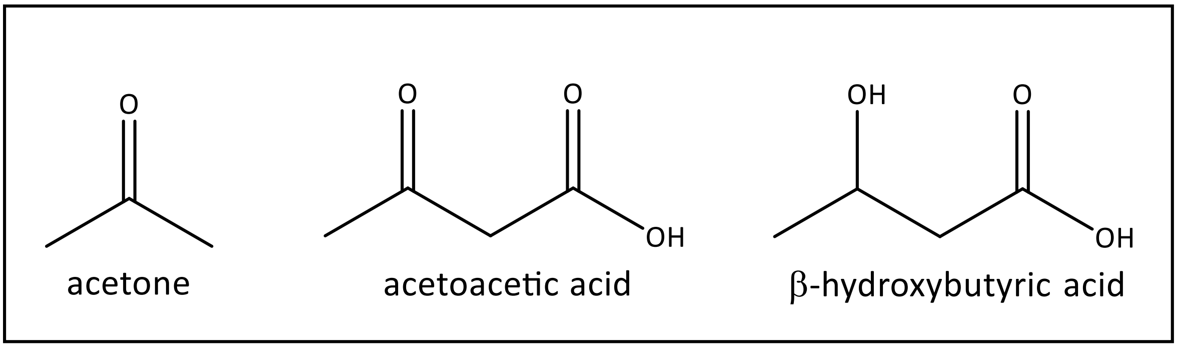

Supplement: Supplementary file 2 [file Data_Sheet_2.PDF]
