# Supplementary figures and images for: A Systematic Review of Intravenous β-Hydroxybutyrate Use in Humans – A Promising Future Therapy?
Source: Front Med (Lausanne). 2021 Sep 21;8:740374. doi: 10.3389/fmed.2021.740374 (PMC8490680; doi:10.3389/fmed.2021.740374)

Figure 3: Flow diagram outlining article identification and selection.

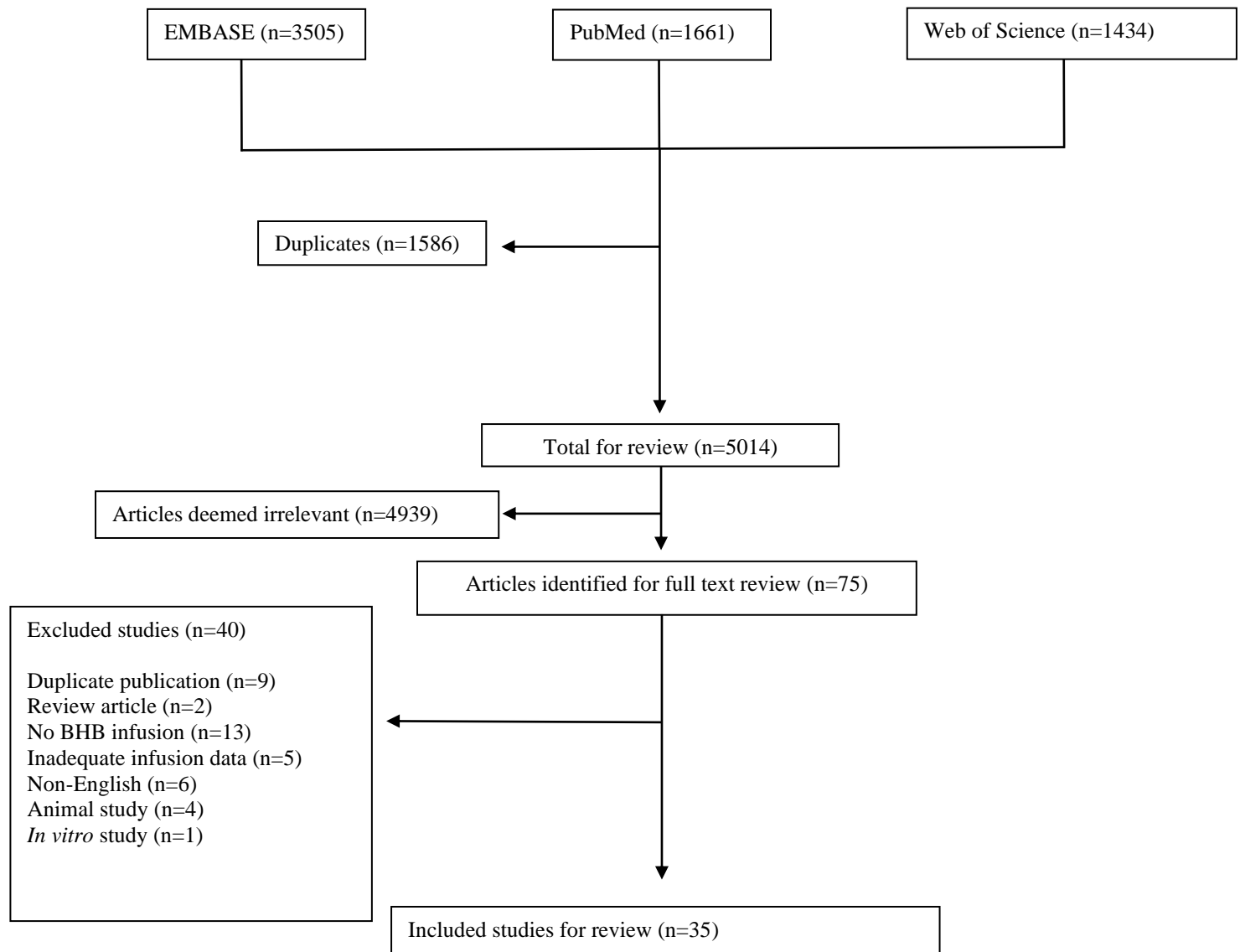

Supplement: Supplementary file 3 [file Data_Sheet_3.PDF]
